# Gamma secretase inhibitors, DAPT and MK0752, exhibit synergistic anticancer effects with cisplatin and docetaxel in 2D and 3D models of breast cancer

**DOI:** 10.55730/1300-0152.2776

**Published:** 2025-10-10

**Authors:** Kübra TELLİ, Johannes GUBAT, Pádraig D’ARCY, Özden YALÇIN ÖZUYSAL

**Affiliations:** 1 Department of Molecular Biology and Genetics, Faculty of Science and Letters, Istanbul Kültür University, İstanbul, Turkiye; 2 Division of Clinical Chemistry and Pharmacology, Department of Biomedical and Clinical Sciences, Linköping University, Linköping, Sweden; 3 Department of Molecular Biology and Genetics, Faculty of Science and Letters, Izmir Institute of Technology, İzmir, Turkiye

**Keywords:** Notch signaling pathway, gamma secretase inhibitors, DAPT, MK0752, mammospheres

## Abstract

**Background/aim:**

Breast cancer remains a major malignancy among women, and severe side effects and the development of acquired drug resistance frequently hinder current therapeutic strategies. The Notch signaling pathway, a key regulator of cell fate, is commonly dysregulated in breast cancer and associated with poor prognosis. Gamma-secretase inhibitors (GSIs) block Notch receptor activation and have shown potential anticancer efficacy. This study aimed to investigate the synergistic activity of two commonly used GSIs, DAPT and MK0752, combined with docetaxel or cisplatin in both 2D and 3D breast cancer models.

**Materials and methods:**

Triple-negative, highly metastatic MDA-MB-231 and ER+/PR+ MCF-7 breast cancer cell lines were treated with DAPT or MK0752 alone or in combination with docetaxel or cisplatin. Drug efficacy and potential synergism were evaluated in 2D monolayer cultures and 3D spheroid models. Sequential treatment strategies were also assessed, where docetaxel or cisplatin was administered prior to GSI exposure.

**Results:**

Both MDA-MB-231 and MCF-7 cell lines exhibited notable sensitivity to DAPT and MK0752 combinations with docetaxel or cisplatin in 2D and 3D cultures. Synergistic enhancement of cytotoxicity was observed, particularly in sequential treatment regimens. Pretreatment with docetaxel or cisplatin followed by GSI exposure demonstrated superior growth inhibition compared with either monotherapy or simultaneous combination treatments.

**Conclusion:**

This study highlights the therapeutic potential of combining GSIs with standard chemotherapeutics to overcome drug resistance in breast cancer. The observed synergy and sequencing effects provide a strong basis for further mechanistic and translational investigations to optimize GSI-based combinational therapy strategies.

## 1. Introduction

Breast cancer remains one of the most malignant cancers among women, accounting for a 6.9% mortality rate worldwide (Sung et al., 2021). Hormone therapy and chemotherapy persist as the primary therapeutic strategies; however, issues such as acquired drug resistance, relapse, and dose-limiting toxicities continue to hinder treatment success. The presence or absence of estrogen (ER), progesterone (PR), and human epidermal growth factor receptor 2 (HER2) defines breast cancer subtypes and guides treatment selection. Notably, triple-negative breast cancer (TNBC), which lacks these receptors, remains unresponsive to hormone or HER2-targeted therapies (Waks and Winer, 2019). Conventional chemotherapies typically target highly proliferative cells but are often constrained by toxicity (Mollen et al., 2018). In contrast, novel targeted therapies aim to modulate dysregulated molecular pathways to counteract long-term cancer adaptation (Harbeck et al., 2019). Combining traditional and targeted therapies can enhance treatment efficacy while mitigating side effects (Ianevski et al., 2020). Among targeted pathways, Notch signaling has garnered considerable attention due to its aberrant activation in various cancers. This pathway regulates key cellular processes, including proliferation, differentiation, apoptosis, and drug resistance, through its downstream effectors (Krzyszczyk et al., 2018). The pathway is activated by five canonical ligands (Jagged 1/2, DLL1/3/4) and four receptors (Notch 1–4), culminating in receptor cleavage by the γ-secretase complex (comprising Presenilin, PEN2, APH1, and Nicastrin) and nuclear translocation of the Notch intracellular domain (NICD) to activate target genes (Bazzoni and Bentivegna, 2019). Inhibition of Notch signaling has been shown to reduce tumor size, metastasis, and relapse in preclinical models (Artavanis et al., 1995). Gamma-secretase inhibitors (GSIs), such as DAPT and MK0752, prevent NICD release and have shown promising outcomes both as monotherapies and in combination with standard chemotherapeutics across various cancer types (Brennan and Klarke, 2013; Takebe et al., 2014). Nevertheless, despite encouraging results, GSI monotherapies often exhibit limited efficacy due to context-dependent Notch pathway activation, off-target effects, and incomplete tumor regression. Additionally, GSIs may induce gastrointestinal toxicity and fail to eradicate cancer stem cell populations when used alone. Combining GSIs with conventional agents such as docetaxel and cisplatin may potentiate cytotoxicity by concurrently targeting proliferative and survival pathways. Notch inhibition has been reported to sensitize tumor cells to DNA-damaging or mitotic agents, providing a rationale for dual-targeted strategies (Schott et al., 2013; Tamagnone et al., 2018; Feng et al., 2019; McCaw et al., 2021). Here, we investigated whether the GSIs DAPT and MK0752 could synergize with docetaxel or cisplatin in breast cancer cells using both monolayer (2D) and mammosphere (3D) models of the MDA-MB-231 (triple-negative) and MCF-7 (ER/PR+) lines. Furthermore, we evaluated whether the sequence of drug administration, sequential versus simultaneous, modulates the efficacy of combination therapy in a subtype-dependent manner. Our study aims to identify optimal therapeutic strategies by integrating targeted inhibition of Notch signaling with established chemotherapeutics to overcome current limitations and improve treatment outcomes.

## 2. Materials and methods

### 2.1. Cell culture conditions

Human TNBC cell line MDA-MB-231 (ATCC HTB26) and ER and PR receptor-positive breast cancer cell line MCF-7 (ATCC HTB22) were used as breast cancer models. They were cultured in Dulbecco’s Modified Eagle Medium (DMEM) (Sigma) supplemented with 1% penicillin/streptomycin (Thermo) and 10% fetal bovine serum (Biological Industries) at 37 °C in 5% CO_2_. Cells, when they reached confluency of 80–90%, were washed with room-temperature phosphate-buffered saline (PBS) and trypsinized (0.05%) for further passaging.

### 2.2. Cell viability assay and half maximal inhibitory concentration (IC_50_) calculations

Stock solutions of DAPT (Cayman), MK0752 (Cayman), and docetaxel (TOCRIS) were prepared in dimethyl sulfoxide (DMSO). Cisplatin (Sigma) was dissolved in 0.9% sodium chloride (NaCl) solution. DMSO and NaCl solvents were included as controls throughout the experiments. IC_50_ dose was determined by using MTT (3-(4,5-dimethylthiazol-2-yl)-2,5 diphenyltetrazolium bromide) cell viability assay. Cells were plated into 96-well plates and treated with increasing doses of DAPT, MK0752, docetaxel and cisplatin (from 1 nM to 100 μM) or respective solvent controls for 24, 48, or 72 h. Cells were then treated with 5 mg/mL of MTT (Amensco) for 4 h, followed by dissolving the tetrazolium salts in 10% sodium dodecyl sulphate and 0.01 M hydrochloric acid. The absorbance values at 570 nm and 650 nm were measured in a microtiter plate reader (Thermo Scientific Multiskan Spectrum). Intrinsic sensitivities are defined as: highly sensitive (IC_50_ < 1 μM), sensitive (IC_50_ = 1–50 μM), resistant (IC_50_ = 50–100 μM), and highly resistant (IC_50_ > 100 μM).

### 2.3. Synergy calculations

Drug combination doses were selected according to each cell line’s IC_50_ value for each drug tested. IC_12.5_, IC_25_, IC_50_, and IC_100_ dose combinations were tested for DAPT and MK0752 with docetaxel or cisplatin. MTT absorbance values of three independent combination conditions were analyzed for synergism by using the Synergy Finder tool (SynergyFinder 2.0 version). The Bliss Independence model was selected to evaluate combinational effects, as it is particularly appropriate for drugs with distinct mechanisms of action and independent activity, such as the combinations tested in this study: GSI with docetaxel or GSI with cisplatin (Ianevski et al., 2020). Independent experimental setups were replicated at least three times. Nontreated controls were used for normalization.

### 2.4. Multidrug treatment strategies

Multidrug treatment was applied using three strategies: single-agent, combinational, and sequential treatments with DAPT, MK0752, docetaxel, and cisplatin. Single-agent treatments involved a single application of each drug, and combinational treatments used two selected drugs applied concurrently (McCaw et al., 2021). A sequential treatment strategy was mimicked by administering a single drug followed by a second drug at the end of a 24-h incubation period (Moo et al., 2018). Treatments were incubated for 72 h, followed by an MTT cell viability assay. Results of a minimum of three independent replicates were analyzed.

### 2.5. Mammosphere formation and analysis

MDA-MB-231 and MCF-7 cells were seeded at 5000 cells per well in a 96-well U-bottom plate and cultured for 3 to 4 days in growth media until spheres formed. Then, IC_100_ of DAPT, MK0752, docetaxel, and cisplatin were applied, and images were captured using Incucyte Cell Analysis Systems for up to 72 h. ImageJ’s SpheroidJ plugin was used to measure sphere sizes. Day 3 values were normalized to Day 0 within each group. Images of a minimum of three independent replicates were analyzed.

### 2.6. Acid phosphatase assay

The viability of mammospheres was assessed by acid phosphatase assay. Mammospheres were treated with p-nitrophenyl phosphate solution in sodium acetate buffer supplemented with Triton-X-100 (0.1%) and incubated at 37 °C for 90 min. The phosphatase reaction was terminated with 0.2% 1N NaOH, and absorbance at 405 nm was measured in a microtiter plate reader (Thermo Scientific Multiskan Spectrum). Each condition was normalized to the respective untreated control.

### 2.7. Statistical analysis

All data were analyzed using GraphPad Prism 9. Circularity measurements were evaluated using one-way ANOVA with appropriate post hoc tests. For experiments involving multiple variables, two-way ANOVA followed by multiple comparisons and paired t-tests was applied. Each condition was normalized to its respective untreated control. Statistical significance was defined as follows: ns (not significant), p > 0.05; *: p < 0.05; **: p < 0.01; ***: p < 0.001; ****: p < 0.0001.

## 3. Results

### 3.1. Intrinsic sensitivity and synergistic profiles of breast cancer cell lines to DAPT, MK0752, cisplatin, and docetaxel

**Figure 1:**
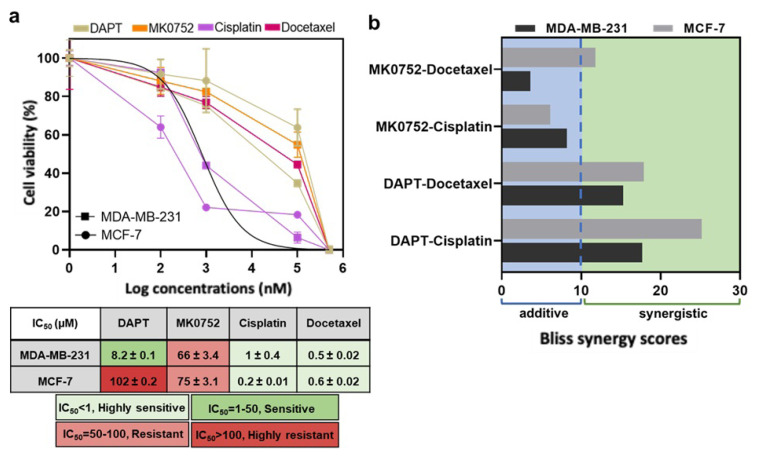
Intrinsic sensitivity and synergy scores of MDA-MB-231 and MCF-7 cells to DAPT, MK0752, docetaxel, and cisplatin treatments. (a) IC_50_ curves and values are colored based on the sensitivity scaling. (b) Bliss scores of DAPT and MK0752 with cisplatin and docetaxel. Error bars represent mean ± SD from three biological replicates.

IC_50_ values of DAPT, MK0752, cisplatin, and docetaxel for MDA-MB-231 and MCF-7 cell lines were determined by MTT cell viability assay. IC_50_ values were calculated as 8.2 μM for DAPT, 66 μM for MK0752, 1.01 μM for cisplatin, and 0.5 μM for docetaxel in MDA-MB-231 cells. IC_50_ values for MCF-7 cells were calculated as 102 μM for DAPT, 75 μM for MK0752, 0.2 μM for cisplatin, and 0.6 μM for docetaxel. The intrinsic toxicity response scale showed that MDA-MB-231 and MCF-7 exhibit high sensitivity to docetaxel and cisplatin and moderate resistance to MK0752. MDA-MB-231 cells were intrinsically sensitive to DAPT, whereas MCF-7 cells exhibited a highly resistant phenotype (Figure 1a).

To verify DAPT and MK0752’s potential synergistic outcome with cisplatin and docetaxel, 25 conditions based on the IC_50_ value range of drugs were arrayed, and cell viability data were plotted into a dose-response matrix to assess Bliss synergy scores of combinations. Each dose for both single-agent and combinational treatments was applied simultaneously, and the incubation period was set to 72 h (Supplementary Figure). To determine synergy scores, the Bliss synergy approach as described by Ianevski et al. (2020) was used. A bliss score less than −10 indicates that two drugs are likely to be antagonistic; from −10 to 10, they are likely to be additive; and greater than 10, they are likely to be synergistic (SynergyFinder 2.0 version) (Ianevski et al., 2020). MDA-MB-231 cell line’s Bliss scores of DAPT combinations with cisplatin were 17.7 and with docetaxel 15.3, whereas MK0752 treatments’ Bliss score with cisplatin was 8.2 and 3.6 for docetaxel combinations. MCF-7 cell line’s Bliss scores of DAPT combinations with cisplatin were 25.2 and with docetaxel 17.9, whereas MK0752 treatments’ Bliss score with cisplatin was 6.1 and 11.8 for docetaxel combinations (Figure 1b). DAPT’s combination with cisplatin and docetaxel showed highly synergistic activity in both cell lines, whereas MK0752 combinations showed only weak synergism. For the following analyses, IC_100_ doses of all drugs were used, as they showed the highest synergistic effect (Supplementary Figure).

### 3.2. Combination of gamma secretase inhibitors with cisplatin and docetaxel decreases breast cancer cell viability in 2D

**Figure 2:**
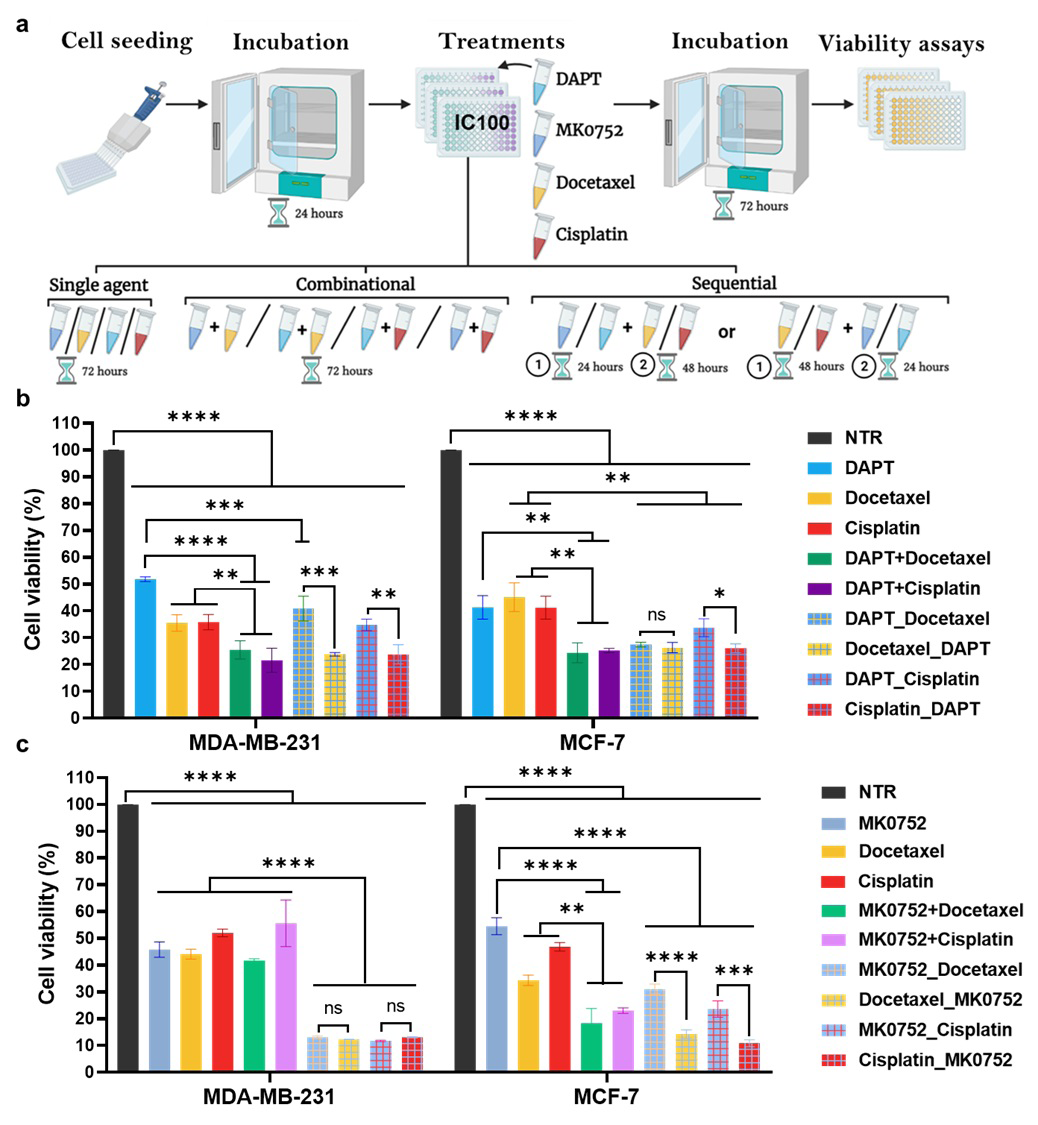
Single-agent, combinational, and sequential treatment strategies with DAPT and MK0752, combined with docetaxel and cisplatin, decrease the viability of MDA-MB-231 and MCF-7 cells. (a) Schematic representation of single-agent, combinational and sequential treatment strategies. MDA-MB-231 and MCF-7 cell viability percentage viabilities of (b) DAPT and (c) MK0752 treatments based on MTT absorbance values. The plus sign (“+”) indicates combinations, and the underscore (“_”) indicates the order of the sequential treatments. Values were normalized to the nontreated (NTR) groups. p > 0.05 as *ns: not significant; *p < 0.05; **p < 0.01; ***p < 0.001; ****p < 0.0001. Statistical significance was determined using one-way ANOVA with appropriate post hoc tests. Error bars represent mean ± SD.

To investigate the effect of different treatment strategies, we applied the drug combinations simultaneously (Figure 2a, combinational) or sequentially, where the first drug (GSI or chemotherapeutic agent) was applied before the second drug (Figure 2a, sequential) and after the incubation period of 72 h, an MTT cell viability assay was performed.

In MDA-MB-231 cells, DAPT, docetaxel, and cisplatin reduced viability by 48.2%, 64.5%, and 64.2%, respectively, compared with the nontreated control. A combination of DAPT with docetaxel or cisplatin showed a significantly stronger effect than the single drug treatment, decreasing viability by 74.6% and 78.5%, respectively (Figure 2b, left). Sequential treatments showed an order-dependent effect. Applying DAPT after an initial docetaxel or cisplatin treatment had a similar effect, with combinational treatment reaching a 76.2% decrease. On the other hand, using DAPT as the initial treatment, followed by docetaxel or cisplatin, showed only a moderate effect (Figure 2b, left). In MCF-7 cells, DAPT, docetaxel, and cisplatin reduced viability by 58.8%, 54.9%, and 58.9%, respectively, compared with the nontreated control. Combinational treatment had a stronger effect, reducing the viability by 75.7% when combined with docetaxel and by 74.8% with cisplatin (Figure 2b, right). In contrast to MDA-MB-231 cells, the effect of sequential treatments was similar to that of the combinational treatment, independent of drug order (Figure 2b, right). In conclusion, simultaneous application of the drugs or applying DAPT after docetaxel or cisplatin has anticancer effects in both breast cancer cell lines.

In contrast to DAPT, the combination of MK0752 with docetaxel or cisplatin did not significantly reduce viability compared with single-drug treatments in MDA-MB-231 cells (Figure 2c, left). However, docetaxel and cisplatin initial treatment, followed by MK0752, decreased viability dramatically by 87%. MK0752 continued by cisplatin or docetaxel treatment resulted in a decrease in viability at a similar rate (Figure 2c, left). In MCF-7 cells, combinational treatment with MK0752, docetaxel, and cisplatin showed a stronger effect, reducing viability by 81.6% and 77%, respectively, compared with single-drug applications, in which DAPT, cisplatin, and docetaxel decreased viability by 45.5%, 65.6%, and 53.2%, respectively (Figure 2c, right). In MCF-7 cells, the strongest effect was achieved in sequential treatments, in which MK0752 treatment after an initial docetaxel or cisplatin treatment decreased viability by 85.8% and 89.2%, respectively (Figure 2c, right). In conclusion, MK0752 was most effective in sequential treatments with docetaxel or cisplatin in breast cancer cell lines. Overall, in 2D, DAPT and MK0752 showed synergistic effects with docetaxel and cisplatin in MDA-MB-231 and MCF-7 breast cancer cell lines, but the most effective treatment strategy varied by cell line and GSI.

### 3.3. DAPT and MK0752 combinations with cisplatin and docetaxel decrease mammosphere size and cell viability

**Figure 3:**
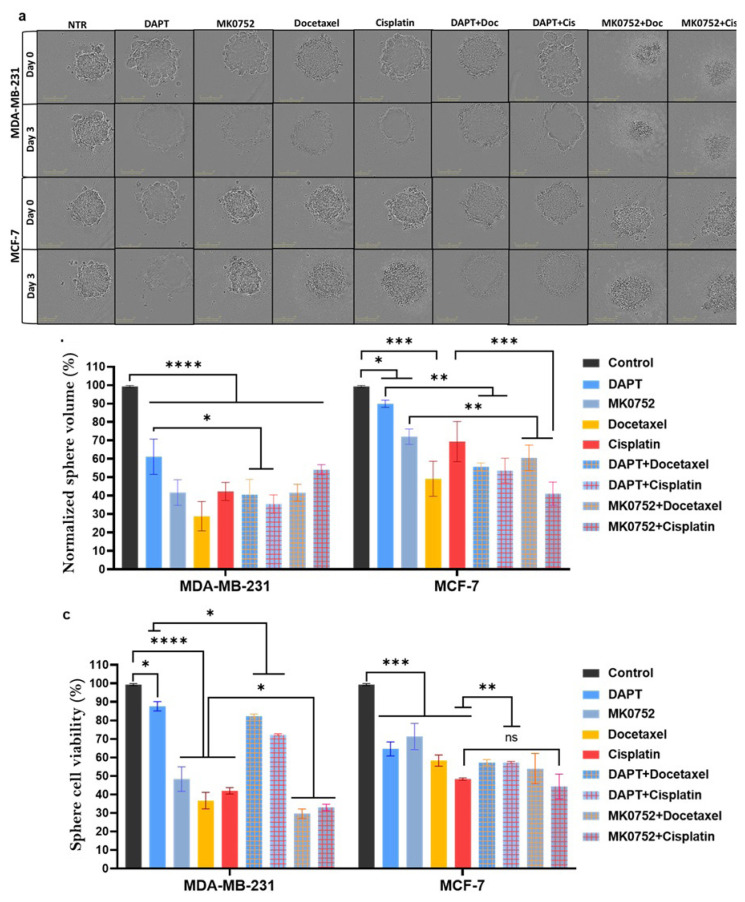
DAPT, MK0752, docetaxel and cisplatin treatments decrease mammosphere size and cell viability in both single-agent and combinational treatments. (a) Sphere images of MDA-MB-231 and MCF-7 cell lines at days 0 and 3, (b) normalized sphere volumes after treatments and (c) sphere cell viability at day 3. *ns: not significant (p > 0.05); *p < 0.05; **p < 0.01; ***p < 0.001; ****p < 0.0001. Statistical significance was determined using one-way ANOVA with appropriate post hoc tests. Error bars represent mean ± SD.

One of the mechanisms by which tumors evade apoptosis and induce drug resistance is the regulation of the microenvironment through hypoxia, pH, and acidity (Hanahan and Weinberg, 2011; Bogdanov et al., 2022). To evaluate the GSI effects, we generated mammospheres to receive more relevant biological readouts that mimic such environmental conditions. MDA-MB-231 and MCF-7 mammospheres were treated with IC_100_ of drugs for 3 days (Figure 3a). Then, the images of spheroids were analyzed to estimate shrinkage in spheroid size, followed by the acid phosphatase assay to assess cell viability.

The size of MDA-MB-231 mammospheres decreased to 61.1%, 41.6%, 28.8%, and 42.2% in response to DAPT, MK0752, docetaxel, and cisplatin treatments (Figure 3b, left). Combinational treatment with docetaxel or cisplatin and DAPT further reduced sphere size, whereas no synergistic effect was observed when they were combined with MK0752 (Figure 3b, left). In parallel with sphere size, the viability of the MDA-MB-231 spheres was also reduced in response to single drug treatments. Furthermore, the combination of MK0752, but not DAPT, showed the strongest effect, with viability reduced to 29.6% with docetaxel and 33% with cisplatin (Figure 3c, left).

In MCF-7 mammospheres, combination treatments reduced both size and viability; however, the reductions were not as strong as in MDA-MB-231 mammospheres. The most effective reduction was achieved with the MK0752 and cisplatin combination, which reached 41.1% in size and 44.3% in viability (Figure 3b and c, right). In conclusion, the effects of DAPT and MK0752 single-agent treatments were amplified when combined with docetaxel and cisplatin, which confirms their synergistic potential in a more relevant tumor model.

## 4. Discussion and conclusion

Over the last decade, GSIs have emerged as promising therapeutics targeting Notch signalling in cancer (Bogdanov et al., 2022). Approximately 52 monotherapy and 19 combination therapy studies have demonstrated GSI-induced reductions in tumor size, invasion, and cancer stem cell renewal. However, only a few compounds, most notably DAPT and MK0752, have progressed into clinical trials (Dai et al., 2018; McCaw et al., 2021). DAPT has shown potential in reversing platinum resistance in ovarian and gastric cancers when combined with cisplatin (Wang et al., 2014; Kato et al., 2018; Soni et al., 2018). Similarly, MK0752 has exhibited synergism with endocrine therapies such as Tamoxifen and Letrozole in hormone receptor-positive breast cancers, with clinical tolerability and preliminary activity reported in NCT00756717^1^. Additionally, in the clinical trial NCT00645333^2^, MK0752 combined with docetaxel achieved a 45% response rate in metastatic breast cancer patients previously treated with chemotherapy, predominantly in ER+/PR+ and HER2− subtypes (Schott et al., 2013). Building on these findings, our study systematically evaluated DAPT and MK0752 as single agents and in combination and sequential regimens with docetaxel or cisplatin, using both 2D and 3D breast cancer models. Importantly, we stratified our analysis by subtype, assessing triple-negative (MDA-MB-231) and hormone receptor-positive (MCF-7) cell lines, an approach not commonly emphasized in prior preclinical reports. Our findings underscore distinct subtype-specific responses to GSI-based regimens and advocate for tailored therapeutic strategies in clinical trial design. Mammosphere assays, which reflect tumor heterogeneity and stemness features in 3D cultures (Dontu, 2003), revealed that both MK0752 and DAPT reduced viability in MDA-MB-231 and MCF-7 cells, with more pronounced effects observed in combination with docetaxel or cisplatin in 2D cultures. In MCF-7 cells, high sensitivity to MK0752-based combinations aligned with clinical observations from NCT00756717, whereas results from MDA-MB-231 cells, which are typically underrepresented in clinical trials, suggest that triple-negative subtypes may benefit from distinct combination strategies. Interestingly, in MDA-MB-231 mammospheres, DAPT combined with either chemotherapeutic showed reduced viability compared with single-agent controls, despite the lack of consistent morphological changes. In contrast, MK0752-based combinations did not produce significant additive effects in 3D, possibly due to saturation of its impact or context-dependent pharmacodynamics. The inconsistencies observed between sphere size and acid phosphatase assay (APA) viability results in 3D models may be attributed to the structural complexity of mammospheres. These contain proliferative outer zones, quiescent middle layers, and necrotic cores, potentially leading to uneven drug penetration and metabolic gradients. This heterogeneity can lead to under- or overestimation of viability, depending on the assay methodology. Additionally, altered pH gradients and acid diffusion within the dense cores may skew APA-based viability assessments. These findings highlight the importance of optimizing drug concentrations specifically for 3D contexts, potentially through IC₁₀₀ determination and interpreting viability metrics with assay-specific limitations in mind.

Sequential treatment experiments were designed to model clinically relevant therapeutic schedules (Stephen and Wrzesinski, 2012; Moo et al., 2018). We found that chemotherapy administered prior to GSI treatment resulted in significantly greater cytotoxicity than the reverse sequence, particularly in triple-negative MDA-MB-231 cells. This pattern held for both DAPT and MK0752 combinations. In MCF-7 cells, MK0752 after docetaxel or cisplatin led to a greater decrease in viability than the simultaneous or reversed sequences, consistent with data from NCT00645333 and suggesting an underutilized therapeutic window. Notably, our findings contrast with previous clinical protocols in which GSIs were administered before chemotherapy, suggesting that treatment order plays a critical role in maximizing efficacy. At the mechanistic level, docetaxel and cisplatin induce cellular stress via mitotic arrest and DNA damage, respectively, which may activate compensatory survival pathways such as Notch, PI3K/AKT, and NF-κB. Administering GSIs after chemotherapy could intercept this adaptive signaling surge by inhibiting NICD production and nuclear translocation, thereby sensitizing damaged cells to apoptosis. Moreover, chemotherapy may increase the expression or accessibility of Notch receptors and ligands, thereby enhancing GSI target engagement. Conversely, GSI pretreatment may blunt baseline Notch activity without suppressing the postchemotherapy rebound, reducing overall efficacy. These sequence-dependent dynamics likely account for the enhanced toxicity observed in our sequential regimens. To validate this hypothesis, future studies should assess NICD levels, downstream Notch targets (such as HES1 and HEY1), and apoptosis markers at defined time points during treatment. MK0752 demonstrated variable efficacy across different breast cancer subtypes and experimental conditions, highlighting its context-dependent pharmacological profile. Differences in response between MDA-MB-231 and MCF-7 cells may be attributed to varying degrees of intrinsic Notch pathway activity, receptor abundance, GSI sensitivity, or drug diffusion barriers in 3D systems. As a substrate-competitive inhibitor, MK0752’s effectiveness also depends on the availability of Notch ligands and potential activation of compensatory pathways. These results reinforce the need to define molecular or phenotypic biomarkers predictive of GSI responsiveness across diverse breast cancer subtypes.

In conclusion, our study demonstrates that DAPT and MK0752 exhibit additive to synergistic antitumor effects when combined with docetaxel or cisplatin across both 2D and 3D breast cancer models. Importantly, the treatment sequence substantially affects therapeutic outcomes, especially in triple-negative cells, which often have limited therapeutic options. The integration of preclinical and clinical data suggests that chemotherapy-induced activation of survival pathways followed by Notch inhibition may offer a more effective approach than current protocols. Future investigations using patient-derived organoids and in vivo models should focus on identifying predictive biomarkers and refining combination regimens to enable precision-based treatment strategies in breast cancer.

## Supplementary Figure

Supplementary Figure Heatmap graphs of drug synergy analysis by the Synergy Finder tool for MDA-MB-231 and MCF-7 cell lines.

## Footnotes

1 ClinicalTrials.gov (2017). NCT00756717 [online]. Website: https://clinicaltrials.gov/show/NCT00756717/ [accessed May 2023].

2 ClinicalTrials.gov (2017). NCT00645333 [online]. Website: https://clinicaltrials.gov/show/NCT00645333/ [accessed May 2023].
