# Supplementary figures and images for: Gamma secretase inhibitors, DAPT and MK0752, exhibit synergistic anticancer effects with cisplatin and docetaxel in 2D and 3D models of breast cancer
Source: Turk J Biol. 2025 Oct 10;49(7):738–45. doi: 10.55730/1300-0152.2776 (PMC12768437; doi:10.55730/1300-0152.2776)

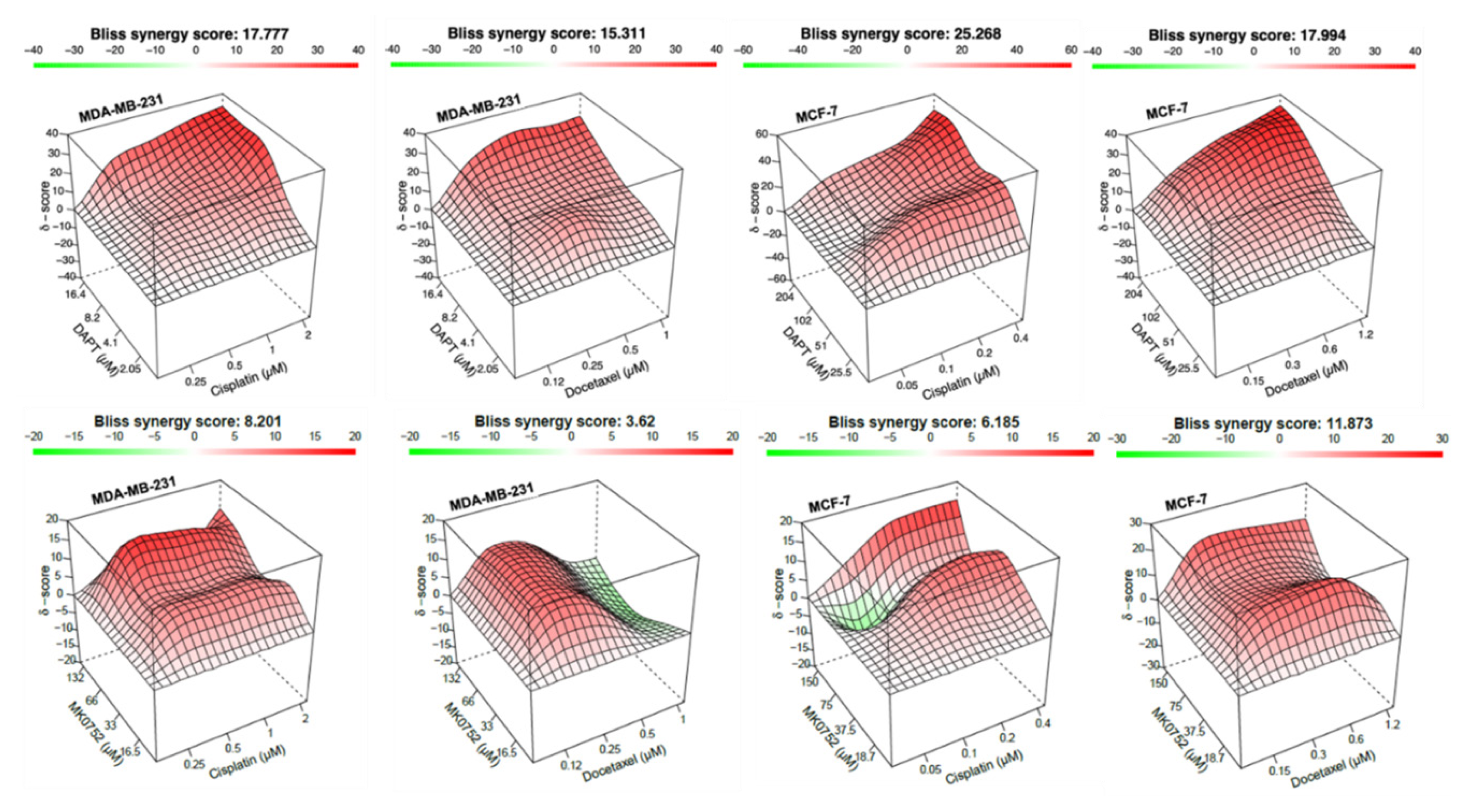

Supplement: Supplementary Figure — Heatmap graphs of drug synergy analysis by the Synergy Finder tool for MDA-MB-231 and MCF-7 cell lines. [file tjb-49-07-738s1.tif]
